# Letter to the Editor in response to the Position statement and best practice recommendations on the imaging use of ultrasound from the European Society of Radiology ultrasound subcommittee

**DOI:** 10.1186/s13244-021-01002-9

**Published:** 2021-05-20

**Authors:** Chris Kalinka, Jeanette Dickson, Richard Evans, Edward Morris, Pamela Parker, Gill Walton, William Ramsden, Gill Harrison

**Affiliations:** 1 Society and College of Radiographers, London, UK; 2 Royal College of Radiologists, London, UK; 3 Consortium for the Accreditation of Sonographic Education Member Organisations, London, UK; 4 Royal College of Obstetricians and Gynaecologists, London, UK; 5 British Medical Ultrasound Society, London, UK; 6 Royal College of Midwives, London, UK

**Keywords:** Ultrasound, Sonographer, Skillmix, Competence

## Abstract

This letter to the editor is in response to the consensus statement from the Ultrasound Subcommittee of the European Society of Radiology, the European Union of Medical Specialists (UEMS) Section of Radiology, and the European Federation of Societies for Ultrasound in Medicine and Biology. It highlights the role of the non-medical sonographer in the UK and the evidence underpinning this safe and effective practice.

Dear Editor in Chief,

With regards to the published consensus statement by the European Society of Radiology (ESR) ultrasound subcommittee [1], we would like to commend the ESR for highlighting the need for high quality education including safe use of the equipment, clinical training, decontamination processes and report writing by the practitioner who undertakes the examination. The importance of saving images to a picture archiving system is also crucial, particularly as imaging is undertaken outside the hospital setting in some countries, to enable review, comparison and audit. We are disappointed to note that the ESR “strongly” recommends ultrasound use by doctors, without mention of the role that radiographers and sonographers have in the provision of high quality ultrasound services around the globe. In some cases, for example the United Kingdom (UK), independent reporting non-medical ultrasound practitioner (sonographer) roles have been in place for decades [2] and there is evidence from across Europe and further afield of the effectiveness of sonographer practice [3–11].

The Society and College of Radiographers (SCoR), the Royal College of Radiologists (RCR), the Consortium for the Accreditation of Sonographic Education (CASE), the Royal College of Obstetricians and Gynaecologists (RCOG), the British Medical Ultrasound Society (BMUS) and the Royal College of Midwives (RCM) firmly believe that team-working with radiologists and other medical and non-medical colleagues provides a safe, effective ultrasound imaging service. In the UK, ultrasound courses outside of medical education programmes can opt to be accredited by CASE. This model helps to ensure a minimum standard of education and curriculum content [12], as recommended by the ESR [1]. We continue to work with stakeholders to lobby for statutory regulation of sonographers. As practice has developed, many sonographers have extended their scope of practice to include interventional procedures, elastography, fetal medicine scans, contrast enhanced ultrasound [13] and leading multidisciplinary team meetings. Some extended roles require statutory regulation, such as the ability to act as referrers for examinations (such as CT scans) that involve the use of ionising radiation. Whilst many sonographers are statutorily regulated as for example, a radiographer (Health and Care Professions Council), nurse or midwife (Nursing and Midwifery Council) there are an increasing number of sonographers who are unable to gain statutory regulation in the UK [14]. Any extension to the scope of practice is developed in conjunction with experienced sonographers and medical practitioners, with competency assessment and on-going audit to evidence high standards of practice by sonographers. We believe that sonographer should be a statutory regulated profession so that individuals are required by law to hold formal ultrasound qualifications in order to practise.

Yours sincerely.

## Data Availability

Not applicable.

## References

[1] European Society of Radiology (ESR) (2020) Position statement and best practice recommendations on the imaging use of ultrasound from the European Society of Radiology ultrasound subcommittee. Insights Imaging 11: 115. 10.1186/s13244-020-00919-x 10.1186/s13244-020-00919-x PMC7652945 33165666

[2] Gibbs V, Edwards H, Harrison G (2017) Independent reporting sonographers: could other countries follow the UK’s lead? Imaging Ther Pract 25–29

[3] Bates JA, Conlon RM, Irving HC (1994). An audit of the role of the sonographer in non-obstetric ultrasound. Clin Radiol.

[4] Cummings J, Edwards H (2013) Local Investigation of outcomes based on ultrasound examinations for suspected inguinal hernia performed by sonographers and radiologists. Ultrasound 21: 12–15. 10.1258/ult.2012.012035

[5] Hakim Z, Ali S, Bale S, Hughes P (2015) A comparative analysis of radiographer versus radiologist in the diagnosis of rotator cuff tears of the shoulder using ultrasound. Orthop Res Rev 7: 135

[6] Hofmann B, Vikestad K (2013) Accuracy of upper abdominal ultrasound examinations by sonographers in Norway. Radiography 19:186–189

[7] Leslie A, Lockyer H, Virjee JP (2000). Who should be performing routine abdominal ultrasound? A prospective double-blind study comparing the accuracy of radiologist and radiographer. Clin Radiol.

[8] Lo RH, Chan PP, Chan LP, Wilde CC, Pant R (2003). Routine abdominal and pelvic ultrasound examinations: an audit comparing radiographers and radiologists. Ann Acad Med.

[9] Riley SJ, Groves CJ, Chandramohan M (2010) Musculoskeletal ultrasound: audit of sonographer reporting. Ultrasound 18: 36–40. 10.1258/ult.2009.009011

[10] Schneider M, Bloesch J, Lombardo P (2014). Abdominal ultrasound referred by the Emergency department: can sonographer findings help guide timely patient management?. Radiography.

[11] Weston MJ, Morse A, Slack NF (1994) An audit of a radiographer based ultrasound service. Br J Radiol 67: 665–667 10.1259/0007-1285-67-799-665 8062007

[12] Consortium for the Accreditation of Sonographic Education (2019) Standards for Sonographic Education. Version 2.0. Consort. Accredit. Sonogr. Educ. Available at: http://www.case-uk.org/information/publications/

[13] Society and College of Radiographers (2019) Ultrasound Workforce UK Census 2019 Available at: https://www.sor.org/sites/default/files/document-versions/2019.7.5_final_scor_ultrasound_workforce_uk_survey_2019_report_v3.pdf. Accessed 10 July 2019

[14] Professional Standards Authority (2019) Report to Health Education England Right-touch assurance for sonographers based on risk of harm arising from practice. Available at: www.professionalstandards.org.uk. Accessed 3 July 2019

